# Food allergy outside of the home: a survey of the Irish family’s experience

**DOI:** 10.1186/2045-7022-5-S3-O27

**Published:** 2015-03-30

**Authors:** Aideen Byrne, Maria Iatan

**Affiliations:** 1 Our Lady’s Children’s Hospital, Crumlin Dublin, Ireland; 2 School of Medicine, Trinity College Dublin, Dublin, Ireland

## Aims

Food allergic children spend much of their time under the supervision of adults other than their parents, in day care, school and other environments. During these times they are at risk of contact with food allergens and of developing reactions. In conjunction with the establishment of a new Dublin allergy clinic, we sought to understand the experience of Irish families, and what type of emergency plans and training was in place for other carers.

## Methods

An ethics committee approved survey was designed and distributed to parents waiting to be seen at a tertiary allergy clinic over a period of 6 weeks as part of a student project.

## Results

117 surveys were collected. 100 were completed by parents of children aged 9.5mths-16yr, regularly being cared for outside of the home. 31 children had experienced allergic reactions in the day care setting and 18 in school. 10 cases had had greater than 10 reactions. Reactions were equivalent between those whose food came from home and those whose food was prepared by others. 49 children carried adrenaline auto-injectors. In 50% of cases parents were the primary trainers for other carers. 30 reported designing their own management plan and 1/3 of these had not sought or received advice from support groups or health care workers in creating it. There was no significant association between having a management plan and allergic reactions. No reports of adrenaline administration were recorded. Of the 17 excluded, 30% reported that they had deferred entrance into child care due to fears of allergic reactions.

## Conclusion

Allergic reactions in food allergic children are common in day care and school environments and may be, more often due to accidental contact with other children’s food than errors in food preparation. There is a need for formal provision of training that is no longer the responsibility of parents. The lack of reports of adrenaline administration may reflect a reluctance to administer.

